# Development of a rule-based natural language processing algorithm to extract sleep information in pediatric primary care patients with a sleep diagnosis

**DOI:** 10.1093/sleepadvances/zpag014

**Published:** 2026-02-13

**Authors:** Joseph W Sirrianni, Ariana Calloway, Syed-Amad Hussain, Hongfang Liu, Christopher W Bartlett, Mattina A Davenport

**Affiliations:** Abigail Wexner Research Institute, Nationwide Children’s Hospital, Columbus, OH, United States; Abigail Wexner Research Institute, Nationwide Children’s Hospital, Columbus, OH, United States; Abigail Wexner Research Institute, Nationwide Children’s Hospital, Columbus, OH, United States; Department of Pediatrics, The Ohio State University, Columbus, OH, United States; Department of Health Data Science and Artificial Intelligence, University of Texas Health Science Center, Houston, TX, United States; Abigail Wexner Research Institute, Nationwide Children’s Hospital, Columbus, OH, United States; Department of Pediatrics, The Ohio State University, Columbus, OH, United States; Abigail Wexner Research Institute, Nationwide Children’s Hospital, Columbus, OH, United States; Department of Pediatrics, The Ohio State University, Columbus, OH, United States

**Keywords:** pediatrics, artificial intelligence, natural language processing, children, adolescents

## Abstract

**Study Objectives:**

The current study employed natural language processing (NLP) to capture multidimensional and transdiagnostic information in pediatric clinical notes. We present a novel, low-resource sleep vocabulary that can be applied to notes to identify pediatric sleep-related mentions automatically.

**Methods:**

Using a combination of existing medical sleep ontologies, interviews with clinicians, and examination of clinical note narratives, we develop a novel vocabulary of pediatric sleep-related terms and phrases that covers both technical terms, abbreviations, and colloquial keywords used in describing pediatric sleep health. We compare our vocabulary against a set of manually annotated clinical notes to determine the effectiveness of our vocabulary for identifying notes with pediatric sleep-related mentions.

**Results:**

Our vocabulary was able to correctly identify clinical notes with pediatric sleep-related mentions with a recall of 0.992 and a precision of 0.852. Most false positives occurred in notes that either explicitly stated no sleep issues or contained text unrelated to patient sleep health (e.g. medication side effects). Among the text spans annotated as sleep-related mentions, 77.1% include at least one keyword from our vocabulary.

**Conclusions:**

Our vocabulary showed excellent performance for identifying pediatric sleep-related mentions at the clinical note level and decent performance for identifying the specific text containing patient mentions. Our low-resource vocabulary, which can be deployed in almost any compute environment, can serve as an identifying first pass over clinical notes to identify which notes or note sections should be further processed by more advanced models or manual annotation review to identify more narrow mentions.

Statement of Significance Extracting multidimensional and transdiagnostic sleep-related information in clinical notes is an essential next step to improve pediatric learning health systems’ cohort identification and harmonization. Without this step, efforts toward automated surveillance of subthreshold symptoms, monitoring sleep disparities in detection and care among pediatric populations, and developing clinical decision support and treatment platforms are limited. Natural language processing (NLP) has emerged as a tool to capture sleep condition information (e.g. insomnia) among adults. Yet, implementation of NLP in pediatrics is emerging. However, our preliminary work shows there are still some challenges with noise and identification before these tools can embed a rule-based approach in the NLP pipeline.

## Introduction

It is established that sleep is a critical factor in youth development; however, many youth across the United States report getting inadequate sleep [1, 2]. Although pediatric primary care (PPC) providers gather sleep-related information during clinical encounters, their time-limited context strongly relies on explicit patient/parent complaints and/or specialty sleep care services to confirm clinical pathways for sleep problems and disorders [3]. The current reliance on patient/parent report and time-intensive evaluations (e.g. polysomnography) results in many patients with subthreshold sleep symptoms (i.e. insufficient sleep durations) being missed or under-detected in PPC [4, 5].

An automated surveillance system could alleviate this issue. By identifying sleep-related mentions contained in clinical notes from patient encounters, a system could be developed to recognize patients who would otherwise be overlooked in the siloed and fragmented systemwide pediatric sleep care continuum (e.g. prevention to specialty services) [6]. This could lead to both improved care for individual patients and an enhanced ability to identify specific sleep cohorts for further study. The first step in developing such a system would be to create a component that could automatically identify and collect pediatric sleep-related mentions in their notes for further examination [7–9].

However, identifying pediatric sleep-related mentions in clinical notes is a complex task for two reasons [10, 11]. First, based upon an initial exploration, pediatric sleep-related mentions can occur in a variety of note types across several siloed departments [5]. In addition, sleep screening protocols vary in a learning health system [3, 4]. Therefore, pediatric sleep-related mentions can appear in almost any clinical context. Second, mentions can be expressed in notes using formal clinical terminology (e.g. “obstructive sleep apnea”), informal clinical terminologies like acronyms (e.g. “osa”), and layperson terminology directly quoting a patient (e.g. “trouble breathing while asleep”) [5, 6]. Therefore, the ideal algorithm to identify mentions needs to (1) address the variability of language in mentions and (2) be able to process a large quantity of documentation by note and department type. Deep learning-based natural language processing (NLP) methods have shown strong performance in extracting clinical information from electronic health records, but they remain difficult to scale in low-resources settings [12, 13]. These approaches typically require large annotated datasets, substantial computational resources, and specialized hardware, all of which may be unavailable to under-resourced health systems [7, 10, 11]. Even if the resources can be procured through external vendors, applying such models to large volumes of clinical text can be expensive and time intensive. Consequently, low-resource methods are required for large-scale screening and initial data extraction tasks.

Alternatively, rule-based approaches are computationally efficient and can be applied over millions of notes without requiring specialized hardware or model fine-tuning [5, 6]. Their transparency and ease of use makes them more applicable and adaptable across institutional contexts. As such, many expert crafted rule-based NLP systems remain widely used [14–16]. Prior work has used rule-based systems to identify adult sleep-related information in clinical notes. For instance, a rule-based text mining algorithm was developed for identifying adult sleep-related information in primary care notes [17]. Past work used a combination of ten sleep-related keywords and structured data rules to identify patients with insomnia [18]. However, to our knowledge, there’s only one publicly available vocabulary to identify adult sleep-related mentions across multiple concepts (e.g. snoring, sleep quality, and daytime sleepiness) [18].

In this exploratory analysis, we propose a rule-based approach to identify clinical notes containing pediatric sleep-related mentions. Our keyword bank incorporates terms across 30 pediatric sleep concepts (e.g. nocturnal enuresis and bedtime struggles). These terms were derived from the Peds B-SATED framework, clinical ontologies, mined from our hospital clinical notes, and qualitative feedback from providers [19, 20]. We apply our keyword bank to a set of annotated clinical notes to identify which notes contain pediatric sleep-related mentions and compare our results with an adult-focused keyword bank [21].

## Methods

We manually annotated 300 well-child visit notes. These were randomly sampled from a cohort of patients ages 2–18 years with at least one sleep diagnosis who received PPC between January 2018 and December 2023. This cohort also received care at least one of the following frontline departments: school-based health, behavioral health, and/or healthy weight clinic. Within our institution’s PPC department, PPC providers are mandated to screen for sleep during well-child visits. In addition, we defined a sleep diagnosed patient as a patient with at least one of the ICD-10 diagnoses listed in Supplementary Table S1. This cohort was selected to ensure our dataset would have a sizable number of pediatric sleep-related mentions, since subthreshold sleep symptoms may be documented inconsistently across the institution’s sleep care continuum. Patient demographic information is reported in Table 1.

**Table 1:** Demographic information of patients in annotation cohort

Category	*n*	%
Patients	297	100
Race		
Hispanic/Latino	39	13
Non-Hispanic Black	95	32
Non-Hispanic White	128	43
Non-Hispanic Multiracial	23	8
Non-Hispanic Other	12	4
Insurance		
Private only	48	16
Public only	196	66
Public and private	51	17
Other	2	1
Notes	300	100
Department		
Behavioral health	106	35
Primary care	174	58
Healthy weight	14	5
School based	6	2
Age at encounter (years) [mean (std)]	10.44 (4.50)	

We developed an annotated dataset of clinical notes that contained various types of pediatric sleep mentions. Two annotators annotated the 300 notes for six dimensions of sleep and three related clinical concepts, for a total of nine mentions of sleep health. The dimensions were: (1) Sleep Behavior Dimension, (2) Sleep Satisfaction Dimension, (3) Alertness and Daytime Sleepiness Dimension, (4) Sleep Timing Dimension, (5) Sleep Efficiency Dimension, (6) Sleep Duration Dimension, (7) Sleep Medication Mentions, (8) Sleep Disorder Mentions, and (9) Sleep Intervention Mentions. The annotations were performed at the text span level, meaning that specific words and sentences were annotated. Annotator disagreements were resolved by consensus between annotators. Their overall agreement across all dimensions was 0.6, using Cohen’s kappa, which indicates moderate agreement [22]. Each individual mention class varied between 0.5 and 0.74 agreement (see Supplementary Table S2 for a full breakdown).

For each note, we assigned an overall label as positive if the note contained at least one pediatric sleep mention or negative otherwise. This work was approved by the NCH IRB. We developed a novel keyword bank, called Davenport and Sirrianni Expanded (DSE), which builds upon our prior vocabulary by leveraging four different sources [5]. First, we used the Peds B-SATED framework and past pediatric sleep literature as the foundation for determining our initial pediatric sleep-related terminology. Second, we consulted several clinical ontologies, including the Medical Subject Headings (MeSH) thesaurus, UMLS, SNOMED-CT, and LOINC, for technical terms related to sleep health. Third, we consulted with clinicians at our hospital about terms they typically use in their documentation, along with any common abbreviations. Lastly, we utilized our in-house clinical note search engine, named DeepSuggest8, to discover similar terms to those identified from the ontologies and clinicians based on word-embedding similarities derived from our actual historic clinical notes [5].

During the keyword development process, we assigned the keywords to 37 distinct high-level categories. Keyword bank concepts would often appear in clinical note text outside of the intended context (e.g. pediatric sleep). For example, seven keywords (e.g. wheezing) would often show up for patients with asthma outside the context window of pediatric sleep. We created a second tier category in the DSE vocabulary that included the seven aforementioned concepts. Thus, our vocabulary had two tiers, tier 1 concepts which were used in our note identification rule-set and tier 2 concepts that were not used for identification due to their ambiguity. The DSE keyword bank associated high-level categories and the category tier assignments are shown in Table 2. We compared two keyword banks in our analysis, Sivarajkumar et al. (SEA) keyword bank (27 words) and our DSE keyword bank (359 words) [5, 6]. We evaluated the predictions from two keyword prediction models, one using the SEA keyword bank and one using our DSE keyword bank. We compared these predictions to our ground truth annotations in Table 3.

**Table 2:** Davenport and Sirrianni Expanded (DSE) keyword bank

High-level category	Keywords	Regular expressions
Tier 1 concepts		
Sleep	“sleep,” “sleeping,” “sleeps,” “slept”	
Insomnia	“insomnia,” “difficulty falling or staying asleep,” “trouble falling or staying asleep”	
Restless leg syndrome	“restless leg,” “leg jerks during sleep”	
Periodic limb movement disorder	“periodic limb movement,” “periodic limb movements,” “leg movement during sleep,” “leg movements during sleep,” “arm movement during sleep,” “arm movements during sleep,” “limb movement,” “limb movements”	
Obstructive sleep apnea	“obstructive sleep apnea,” “sleep apnea,” “sleep disordered breathing,” “apnea,” “osa,” “stops breathing at night,” “gasps at night,” “gasping at night,” “short of breath at night,” “unusual breathing patterns at night,” “sdb,” “breathing pauses”	
Nocturnal enuresis	“nocturnal enuresis,” “bedwetting,” “nighttime urinary incontinence”	
Narcolepsy	“narcolepsy,” “cataplexy,” “paroxysmal sleep,” “narcoleptic,” “gelineau’s syndrome”	
Hypersomnia	“hypersomnia,” “sleeps too much,” “slept too much,” “excessive sleep,” “excessive sleeping,” “hypersomnolence,” “long sleep,” “sleeps a lot,” “slept a lot,” “sleeping a lot,” “sleeps more,” “sleeping more,” “oversleep,” “oversleeps,” “oversleeping”	“up to \d + hours,” “more than \d + hours”
Parasomnia	“parasomnia,” “sleep paralysis,” “night terrors,” “confusional arousals,” “sleep terror,” “sleep terrors”	
Sleep-related movement disorder	“sleepwalking,” “sleepwalk,” “sleepwalks,” “sleepwalked,” “acting out dreams,” “acting out dream,” “sleep arousal disorder,” “sleep wake transition disorder,” “sleep talking,” “sleep talk,” “sleep talks,” “sleep head banging,” “sleep related movement disorder”	
Bruxism	“bruxism,” “childhood sleep bruxism,” “nocturnal bruxism,” “sleep bruxism,” “nocturnal teeth grinding disorder,” “teeth grinding at night,” “teeth grinding while sleep,” “teeth grinding,” “grinds teeth,” “grinding teeth”	
Circadian rhythm disorder	“circadian rhythm,” “circadian rhythm disorder,” “delayed sleep phase syndrome,” “delayed sleep,” “sleeps late,” “sleeping late,” “slept late,” “sleeps early,” “sleeping early,” “slept early,” “delayed bedtime,” “bedtime delayed,” “delayed sleep phase,” “advanced sleep phase,” “sleep wake schedule disorder,” “shift worker sleep disorder,” “nonorganic sleep wake cycle disorder,” “non 24 hour sleep wake disorder”	
Sleep locations	“sleeps on couch,” “sleeps on a couch,” “sleeping on a couch,” “sleep in bed,” “sleeps in bed,” “sleeping in a bed,” “sleeps in bedroom,” “sleeping in a bedroom,” “sleeps on bus,” “sleeping on the bus,” “sleeps in car,” “sleeping in the car,” “sleeps in class,” “sleeping in class,” “naps on bus,” “naps on the bus,” “naps at school,” “naps in class,” “naps in car”	
Sleepiness	“sleepiness,” “sleepy,” “sleepier,” “drowsy,” “drowsier,” “drowsiness,” “somnolence,” “excessive sleepiness during the day,” “sleeps during the day,” “doze,” “dozes,” “dozing,” “dozed,” “drowsiness,” “falls asleep in class,” “staying awake”	
Fatigue	“tired,” “fatigue,” “fatigued,” “low energy,” “low-energy,” “no energy”	
Sleep schedule	“bedtime,” “waketime,” “bedtime routine,” “nighttime routine,” “morning routine,” “inconsistent bedtime,” “sleep starts,” “goes to bed,” “wake,” “wakes,” “waking,” “wakes up at,” “school starts at,” “sleeps in,” “gets on bus at,” “bedtime schedule,” “overslept”	
Falling asleep	“falling asleep,” “sleep latency,” “difficulty getting to sleep,” “sleep onset latency,” “difficulty falling asleep,” “trouble falling asleep,” “up at night,” “staying up,” “stays up”	
Difficulty waking	“difficulty waking,” “inability to wake,” “wakefulness”	
Awakenings	“early morning waking,” “early morning awakening,” “early waking,” “wakes early,” “wakes up early,” “waking up early,” “difficulty staying asleep,” “trouble staying asleep,” “awakening,” “awakenings,” “nighttime awakening,” “nighttime awakenings,” “broken sleep,” “waking up,” “wakes up,” “night wake,” “often awake,” “awakening early,” “up during night,” “waking up in the middle”	
Sleep duration	“sleep deprivation,” “insufficient sleep,” “inadequate sleep,” “sleep insufficiency,” “sleep insufficiencies,” “sleep debt,” “sleep duration,” “short sleep,” “short sleeping,” "lack of adequate sleep,” “not getting enough sleep,” “sleep deficit,” “getting enough sleep,” “sleep quantity,” “total sleep time,” “sleepless,” “sleeplessness,” “no sleep,” “inability to sleep,” “unable to sleep”	“less than \d + hours,” “sleep \d + hours”
Sleep quality	“poor sleep,” “poor sleep pattern,” “sleep quality,” “sleep disorder,” “sleep problem,” “sleeping problems,” “trouble sleep,” “trouble sleeping,” “problem sleeping,” “sleep issue,” “sleep issues,” “sleep difficulties,” “sleep difficulty,” “difficulty sleeping,” “difficulties sleep,” “difficulties sleeping,” “problems with sleeping,” “impaired sleep,” “fair sleep quality,” “bad sleep quality,” “tosses and turns in sleep”	
Restless sleep	“restless sleep,” “sleep restless,” “restless sleeping”	
Snoring	“snore,” “snores,” “snoring,” “snoring symptoms”	
Use of medication or supplements to aid sleep	“sleep aid,” “sleeping aids,” “melatonin,” “taking for sleep,” “sleeping pills,” “sleep supplements,” “hypnotics,” “bendryl,” “tylenol pm,” “nyquil,” “chamomile tea,” “lavender,” “valerian,” “atarax,” “tenex,” “clonidine,” “diazepam,” “clonazepam,” “chloral hydrate,” “ambien,” “sonata,” “tricyclics,” “ssris,” “trazadone,” “remeron,” “phenobarbital,” “risperdal,” “topamax”	
Sleep disturbances	“waking during night,” “wakes up at night,” “waking up a night,” “sleep disturbance,” “sleep disturbances,” “disturbance in sleep,” “disturbances in sleep,” “disturbed sleep,” “interrupted sleep,” “interrupting sleep,” “sleep disturbed,” “sleep pattern disturbance,” “sleep disturbance,” “sleep disturbances,” “disturbance in sleep,” “can’t sleep,” “can not sleep,” “can not sleep at all,” “can’t sleep at all,” “sleep fragmentation,” “fragmented sleep,” “trouble sleeping,” “nocturnal agitation”	“\w + keeping \w + up”
Napping	“nap,” “naps,” “napping”	
Sleep hygiene	“sleep hygiene,” “sleep habit,” “sleep habits,” “sleeping habit,” “sleeping habits,” “excessive screentime,” “uses screens at night,” “using screens at night,” "eats late,” “eating late,” “uses electronics at night,” “using electronics at night,” “conflict at bedtime,” “plays at night,” “caffeine”	“doing \w + at night”
Dreams	“nightmare,” “nightmares,” “dream,” “dreams,” “dreaming,” “bad dreams,” “bad dream,” “vivid dreams,” “vivid dreaming,” “vivid dream”	
Bedtime struggles	“bedtime struggle,” “bedtime struggles,” “bedtime resistance,” “bedtime battle”	
Surgery	“tonsillectomy,” “adenoidectomy”	
Tier 2 concepts		
Wheezing	“wheeze,” “wheezing,” “wheezes”	
Hyperactive	“hyper,” “hyperactive,” “hyperactive behavior,” “hyperactive behaviors”	
Dizziness	“dizziness”	
Daytime mood	“irritable,” “grouchy,” “irritability,” “frustrated,” “agitated”	
Tense	“tense sleep,” “tense sleeping,” “trouble winding down at night”	
Inattention	“inattentive,” “troubling focusing,” “poor focus,” “difficulty focusing,” “alertness,” “staying alert”	
Nighttime anxiety	“worries at night,” “worry at night,” “worrying at night,” “anxious at night,” “overthinks at night,” “thoughts at night,” “thinking at night,” “thoughts at bedtime,” “thinking at bedtime,” “anxiety at bedtime”	

**Table 3:** Confusion matrix for the DSE and SEA vocabularies and their precision, recall, and F1-scores

SAE vocabulary			
	Predicted positive	Predicted negative	Total
True positive	209	35	244
True negative	35	21	56
Total	244	56	300
DSE vocabulary			
	Predicted positive	Predicted negative	Total
True positive	242	2	244
True negative	42	14	56
Total	284	16	300
Vocabulary	Precision	Recall	F1-score
SAE	0.857	0.857	0.857
DSE	0.852	0.992	0.917

The software was written in Python and ran in a Linux environment. The code utilized the FlashText library, which implements a variation on the Aho-Corasick algorithm, for string searching and the re package for regular expressions [23]. The DSE vocabulary and code is available at https://github.com/jsirrianni-NCH/rule-based-nlp-sleep-info-pediatric. For our results, we report precision (i.e. the proportion of true positives identified by the keywords out of all instances containing a keyword), recall (i.e. the proportion of true positives identified by the keywords out of all the true positives in the dataset), and F1-score (i.e. the harmonic mean of precision and recall).

## Results

Overall, there were 244 clinical notes containing pediatric sleep-related mentions and 56 notes containing no mentions. The DSE model had a total of two false negatives and 42 false positives, while SEA had 35 false negatives and 35 false positives. The DSE model had a much higher recall (0.992 vs 0.857) while having a comparable precision (0.852 vs 0.857). This difference resulted in a 0.06 difference in F1-score, driven by the increased recall. Table 4 shows the total percentage of tagged pediatric sleep-related category spans in the notes containing at least one keyword from DSE. Across all tags, 77.2% contained at least one keyword from DSE. For each individual tag category, the keyword occurrence ranged from 88.7% (sleep satisfaction) to 54.2% (sleep behaviors). “Sleep” was the most commonly occurring keyword, appearing in 80.7% of all notes with a mention; however, it was also the most common keyword occurring in the false positives, appearing in 71.4% of all false positives. The other keywords had much lower false positive rates but occurred in fewer of the notes. A breakdown of the top 10 appearing keywords across the dataset and in false positives are in Supplementary Tables S3 and S4.

**Table 4:** Tagged spans containing a DSE keyword (tier 1) by dimension type

Tag name	Total tags	Tags containing keyword	Accuracy (%)	Most present keyword (Total tags)
ALL TAGS	1156	892	77.2	Melatonin (101)
Alertness/Day time sleepiness	143	102	71.3	Fatigue (41)
Sleep satisfaction	124	110	88.7	Sleep (38)
Sleep medications	199	162	81.4	Melatonin (101)
Sleep disorder	204	169	82.8	Insomnia (44)
Sleep intervention	99	83	83.8	Sleep (53)
Sleep timing	87	50	57.5	Bedtime (13)
Sleep behavior	96	52	54.2	Sleep (14)
Sleep efficiency	164	131	79.9	Sleep disturbance (37)
Sleep duration	40	33	82.5	Sleep (18)

## Discussion

Rule-based NLP models are easily deployable but lack the contextual understanding needed to determine whether a keyword is used in a clinically valid way [10, 11, 19]. This limitation is evident with terms like “sleep,” which appear in both true positives and false positives due to partial negation (e.g. “patient doesn’t snore often”), speculation (e.g. “patient thinks they might snore”), non-patient references (e.g. “snoring runs in the family”), and other contextual variability, making it difficult to achieve both high recall and high precision. Although large language models can better resolve contextual validity, they are computationally expensive and slow [12, 13]. Therefore, we developed an NLP pipeline that uses a rule-based model as an initial low-resource filter to identify clinical notes likely to contain pediatric sleep-related mentions.

Several limitations of this study should be noted. First, our dataset was derived from a single pediatric institution, which may bias the vocabulary toward the specific health system level characteristics, documentation styles, templates, and jargon used by providers at this site. Second, by sampling patients with an existing sleep diagnosis, our cohort likely has a higher prevalence of pediatric sleep-related mentions than a general primary care population. Consequently, the performance metrics reported here may not fully generalize to undiagnosed populations or other screening contexts (e.g. dental and specialty clinics) where sleep mentions are sparser. Future research is needed to validate this vocabulary systemwide and across multi-institutional datasets, with different ratios of specialty to general clinics and within general pediatric cohorts to ensure its robustness and broader applicability in diverse clinical settings.

In conclusion, we present a preliminary keyword bank for pediatric sleep-related mentions and demonstrate its use within an efficient note classification system. When compared with prior work, our vocabulary increases recall by 13.5% while maintaining precision, supporting its role as an initial step toward accurately and efficiently identifying pediatric sleep-related content in clinical notes at scale [21]. Beyond its immediate research utility, the DSE vocabulary has clear potential applications in clinical operations and quality improvement (QI). For example, in QI initiatives, the algorithm could be used to audit well-child visit documentation and quantify how often sleep-related concerns are recorded, thereby identifying gaps in screening and documentation practices [24]. For cohort identification, the tool could surface patients with sleep-related concerns who lack formal ICD-10 diagnoses, enabling more comprehensive registries for population-level sleep health management [25]. Finally, the low-compute nature of the vocabulary makes it well suited for real-time integration into the electronic health record as a first-pass filter that flags charts containing sleep mentions and triggers downstream best practice advisories [26]. Future work will focus on piloting and evaluating these workflows to ensure they are clinically actionable and do not add to provider burden.

## Supplementary Material

piag007_Supplemental_Files

## Data Availability

Patient data are not available to be shared. Vocabulary set and code available at: https://github.com/jsirrianni-NCH/rule-based-nlp-sleep-info-pediatric.

## Preprint repositories

This manuscript was previously made available as a preprint in medRXiv (DOI: https://doi.org/10.1101/2025.05.31.25328640).
