# Supplementary material for: Development of a rule-based natural language processing algorithm to extract sleep information in pediatric primary care patients with a sleep diagnosis
Source: Sleep Adv. 2026 Feb 13;7(1):zpag014. doi: 10.1093/sleepadvances/zpag014 (PMC12920604; doi:10.1093/sleepadvances/zpag014)
Supplement: piag007_Supplemental_Files [file piag007_supplemental_files.zip › Microneedle Placebo Patch Supplement 23DEC2025_piag007.docx]

**Supplementary Material for:**

**Safety, Reactogenicity, and Acceptability of a Placebo Dissolving Microneedle Patch in Infants and Children**

Carol M. Kao, Christina A. Rostad, Peggy Kettle, Ashley Tippett, Jumi Yi, Inci Yildirim, Kathy Stephens, Chelsea Korski, Brian P. Pollack, Mark R. Prausnitz, Devin V. McAllister, Sebastien Henry, Nadine Rouphael, Evan J. Anderson

**Supplementary Information:**

**Contents**

[**Supplementary Methods** 3](#_Toc217897690)

[**Subject Inclusion Criteria** 3](#_Toc217897691)

[**Subject Exclusion Criteria** 3](#_Toc217897692)

[**Supplementary Table 1. Adverse Event Grading** 4](#_Toc217897693)

[**Supplementary Results** 5](#_Toc217897694)

[**Supplementary Table 2: Solicited Symptoms by Severity and Placebo dMNP** 5](#_Toc217897695)

[**Supplementary Table 3: Parental Acceptability Survey Results: Perception of Placebo dMNP Experience (dMNP #1)** 6](#_Toc217897696)

[**Supplementary Table 4: Parental Acceptability Survey Results: Perception of Placebo dMNP Experience (dMNP #2)** 9](#_Toc217897697)

[**Supplementary Table 5: Parental Acceptability Survey Results: Perception of Placebo dMNP Experience (dMNP #3)** 12](#_Toc217897698)

[**Supplementary Table 6: Parental Acceptability Survey Results: Perception of Placebo dMNP Experience at Final Study Visit (dMNPs #1, #2, #3)** 15](#_Toc217897699)

[**Supplementary Table 7: Overall Experience with the Placebo dMNP** 17](#_Toc217897700)

# **Supplementary Methods**

## **Subject Inclusion Criteria**

A subject must meet all the following criteria to be eligible to participate in this study:

1. LAR provides written informed consent prior to any study procedures being performed.
2. Subject is between the ages of 6 weeks and 24 months, inclusive, on the day of signing informed consent.
3. Subject is in good health as determined by vital signs, medical history, and a targeted physical examination.
4. LAR is able to understand and comply with required study procedures.

## **Subject Exclusion Criteria**

A subject will not be eligible to participate in this study if any the following criteria apply:

1. Subject has an acute illness with fever (temperature >100.4 **°**F) within 72 hours prior to enrollment.
2. Subject has a known chronic medical problem.
3. Subject has known immunosuppression due to underlying illness or treatment, including (but not limited to): Human Immunodeficiency Virus (or birth to a HIV-positive mother), hepatitis B or C; organ transplant; active cancer or any history of hematologic cancer; receipt of chemotherapy or radiation therapy; congenital immunodeficiency, anatomical or functional asplenia.
4. Subject has used long-term* high-dose** oral or parenteral glucocorticoids, or high-dose inhaled steroids.***

** Long term is defined as taken for 2 weeks or more in total at any time during the past 2 months.*

*** High dose defined as prednisone ≥ 20 mg total daily dose, or equivalent dose of other glucocorticoids.*

**** High dose defined as >800 mcg/day of beclomethasone dipropionate or equivalent.*

1. Subject has a history of an underlying skin condition (e.g., eczema, atopic dermatitis) or an open lesion (e.g., laceration, abrasion), scar, or rash in the areas of the planned microneedle patch administration which will interfere with the assessment of reactogenicity.
2. Subject or family members have a history of keloid formation.
3. Subject has any condition that, in the opinion of the investigator, may put the subject at increased risk of harm, may cause the subject to be unable to meet the requirements or might otherwise interfere with evaluations required by the study.
4. Subject has received any experimental products within 30 days before study entry or plan to receive experimental products at any time during the study.
5. Subject has received a vaccine within 7 days of enrollment or plans to receive a vaccine within 7 days after enrollment.
6. Subject has previously received immunoglobulin or blood products.

## **Supplementary Table 1. Adverse Event Grading**

| **Grade** | **0** | **1** | **2** | **3** |
| --- | --- | --- | --- | --- |
| **Local Reactogenicity** | | | | |
| **Induration/ Swelling** | None to less than 2.5 cm | 2.5 – 5 cm and does not interfere  with activity | 5.1 – 10 cm or  interferes with  activity | > 10 cm or  prevents daily  activity, erosion, or ulceration |
| **Erythema** | None to less than 2.5 cm | 2.5 – 5 cm | 5.1 – 10 cm | > 10 cm, erythroderma, or a diffuse rash >15% of the body surface area |
| **Ecchymosis** | None | 2.5 – 5 cm | 5.1 – 10 cm | > 10 cm |
| **Tenderness** | None | Mild discomfort to touch | Discomfort with movement | Significant  discomfort at rest |
| **Pain** | None | Does not interfere  with activity | Repeated use of non-narcotic pain reliever > 24 hours or interferes with activity | Any use of narcotic pain reliever or prevents daily  activity |
| **Pruritus (Itching)** | None | Mild | Moderate itching; limiting instrumental activities of daily living | Severe itching; limiting self-care activities of daily living |
| **Systemic Reactogenicity** | | | | |
| **Irritability (Fussiness)** | None | No interference with  activity | Some interference  with activity | Significant;  prevents daily  activity |
| **Lethargy (Drowsiness)** | None | No interference with  activity | Some interference  with activity | Significant;  prevents daily  activity |
| **Decreased appetite** | None | No interference with  activity | Some interference  with activity | Significant;  prevents daily  activity |
| **Vomiting** | None | No interference  with activity | Some interference  with activity | Prevents daily  activity, requires  outpatient IV  hydration |
| **Fever** | None | 100.4 – 101.1 °F | 101.2 – 102.1 °F | >102.1 °F |
| **Unsolicited Adverse Events** | | | | |
| **Other Events** | None | Mild (present but easily tolerated) | Moderate (able to tolerate routine activity with effort) | Severe (unable to continue routine activity) |

# **Supplementary Results**

## **Supplementary Table 2: Solicited Symptoms by Severity and Placebo dMNP**

| **Symptom** | **Maximum Severity** | **Patch #1 (n=25)** | **Patch #2 (n=23)** | **Patch #3 (n=23)** |
| --- | --- | --- | --- | --- |
| ANY Solicited LOCAL SYMPTOM | Grade 0 | 25 (100.0) | 23 (100.0) | 23 (100.0) |
|  | Grade 1 | 0 (0.0) | 0 (0.0) | 0 (0.0) |
|  | Grade 2 | 0 (0.0) | 0 (0.0) | 0 (0.0) |
|  | Grade 3 | 0 (0.0) | 0 (0.0) | 0 (0.0) |
| Pain | Grade 0 | 25 (100.0) | 23 (100.0) | 23 (100.0) |
|  | Grade 1 | 0 (0.0) | 0 (0.0) | 0 (0.0) |
|  | Grade 2 | 0 (0.0) | 0 (0.0) | 0 (0.0) |
|  | Grade 3 | 0 (0.0) | 0 (0.0) | 0 (0.0) |
| Erythema (Measurement) | Grade 0 | 25 (100.0) | 23 (100.0) | 23 (100.0) |
|  | Grade 1 | 0 (0.0) | 0 (0.0) | 0 (0.0) |
|  | Grade 2 | 0 (0.0) | 0 (0.0) | 0 (0.0) |
|  | Grade 3 | 0 (0.0) | 0 (0.0) | 0 (0.0) |
| Ecchymosis (Measurement) | Grade 0 | 25 (100.0) | 23 (100.0) | 23 (100.0) |
|  | Grade 1 | 0 (0.0) | 0 (0.0) | 0 (0.0) |
|  | Grade 2 | 0 (0.0) | 0 (0.0) | 0 (0.0) |
|  | Grade 3 | 0 (0.0) | 0 (0.0) | 0 (0.0) |
| Induration/swelling | Grade 0 | 25 (100.0) | 23 (100.0) | 23 (100.0) |
|  | Grade 1 | 0 (0.0) | 0 (0.0) | 0 (0.0) |
|  | Grade 2 | 0 (0.0) | 0 (0.0) | 0 (0.0) |
|  | Grade 3 | 0 (0.0) | 0 (0.0) | 0 (0.0) |
| Induration/swelling (Measurement) | Grade 0 | 25 (100.0) | 23 (100.0) | 23 (100.0) |
|  | Grade 1 | 0 (0.0) | 0 (0.0) | 0 (0.0) |
|  | Grade 2 | 0 (0.0) | 0 (0.0) | 0 (0.0) |
|  | Grade 3 | 0 (0.0) | 0 (0.0) | 0 (0.0) |
| Pruritis | Grade 0 | 25 (100.0) | 23 (100.0) | 23 (100.0) |
|  | Grade 1 | 0 (0.0) | 0 (0.0) | 0 (0.0) |
|  | Grade 2 | 0 (0.0) | 0 (0.0) | 0 (0.0) |
|  | Grade 3 | 0 (0.0) | 0 (0.0) | 0 (0.0) |
| Tenderness | Grade 0 | 25 (100.0) | 23 (100.0) | 23 (100.0) |
|  | Grade 1 | 0 (0.0) | 0 (0.0) | 0 (0.0) |
|  | Grade 2 | 0 (0.0) | 0 (0.0) | 0 (0.0) |
|  | Grade 3 | 0 (0.0) | 0 (0.0) | 0 (0.0) |
| Any Solicited SYSTEMIC Symptom | Grade 0 | 16 (64.0) | 17 (73.9) | |
|  | Grade 1 | 7 (28.0) | 4 (17.4) | |
|  | Grade 2 | 2 (8.0) | 1 (4.3) | |
|  | Grade 3 | 0 (4.0) | 1 (4.3) | |
| Fever | Grade 0 | 24 (96.0) | 23 (100.0) | |
|  | Grade 1 | 1 (4.0) | 0 (0.0) | |
|  | Grade 2 | 0 (0.0) | 0 (0.0) | |
|  | Grade 3 | 0 (0.0) | 0 (0.0) | |
| Irritability | Grade 0 | 18 (72.0) | 18 (78.3) | |
|  | Grade 1 | 5 (20.0) | 3 (13.0) | |
|  | Grade 2 | 2 (8.0) | 1 (4.3) | |
|  | Grade 3 | 0 (0.0) | 1 (4.3) | |
| Lethargy | Grade 0 | 23 (92.0) | 23 (100.0) | |
|  | Grade 1 | 2 (8.0) | 0 (0.0) | |
|  | Grade 2 | 0 (0.0) | 0 (0.0) | |
|  | Grade 3 | 0 (0.0) | 0 (0.0) | |
| Decreased appetite | Grade 0 | 25 (100.0) | 22 (95.7) | |
|  | Grade 1 | 0 (0.0) | 1 (4.3) | |
|  | Grade 2 | 0 (0.0) | 0 (0.0) | |
|  | Grade 3 | 0 (0.0) | 0 (0.0) | |
| Vomiting | Grade 0 | 23 (92.0) | 23 (100.0) | |
|  | Grade 1 | 2 (8.0) | 0 (0.0) | |
|  | Grade 2 | 0 (0.0) | 0 (0.0) | |
|  | Grade 3 | 0 (0.0) | 0 (0.0) | |

**Parent Perception of Microneedle Patch Experience**
Parental perception of symptoms in their children after MNP placement was overall minimal, although surveyed responses differed somewhat from the solicited AEs reported on the memory aids. Since this information was usually based upon parental recall rather than on real-time observation (memory aids), these are detailed here separately.

## **Supplementary Table 3: Parental Acceptability Survey Results: Perception of Placebo dMNP Experience (dMNP #1)**

| **Symptom** | **Post-dMNP**  **Day 1**** | **Post-dMNP**  **Day 2** | **Post-dMNP**  **Day 8** | **Final Visit***** |
| --- | --- | --- | --- | --- |
| Since the time your child received a vaccine patch, has your child experienced any lasting pain resulting from the vaccine patch and for how long^1^: |  |  |  |  |
| For how many **minutes** did the lasting pain last? |  |  |  |  |
| None | 24 (96.0) |  |  |  |
| < 1 minute | 1 (4.0) |  |  |  |
| 1 – 5 minutes | 0 (0.0) |  |  |  |
| 6 – 10 minutes | 0 (0.0) |  |  |  |
| 11 – 30 minutes | 0 (0.0) |  |  |  |
| Ongoing | 0 (0.0) |  |  |  |
| For how many **hours** did the lasting pain last? |  |  |  |  |
| None |  | 25 (100.0) |  |  |
| < 1 hour |  | 0 (0.0) |  |  |
| 1 – 4 hours |  | 0 (0.0) |  |  |
| 5 – 12 hours |  | 0 (0.0) |  |  |
| 13 – 24 hours |  | 0 (0.0) |  |  |
| Ongoing |  | 0 (0.0) |  |  |
| For how many **days** did the lasting pain last? |  |  |  |  |
| None |  |  | 25 (100.0) | 25 (100.0) |
| < 1 day |  |  | 0 (0.0) | 0 (0.0) |
| 1 – 2 days |  |  | 0 (0.0) | 0 (0.0) |
| 3 – 4 days |  |  | 0 (0.0) | 0 (0.0) |
| 5 – 7 days |  |  | 0 (0.0) | 0 (0.0) |
| Ongoing |  |  | 0 (0.0) | 0 (0.0) |
| For how many **minutes** did the swelling/redness last? |  |  |  |  |
| None | 9 (36.0) |  |  |  |
| < 1 minute | 2 (8.0) |  |  |  |
| 1 – 5 minutes | 0 (0.0) |  |  |  |
| 6 – 10 minutes | 0 (0.0) |  |  |  |
| 11 – 30 minutes | 4 (16.0) |  |  |  |
| Ongoing | 10 (40.0) |  |  |  |
| For how many **hours** did the swelling/redness last? |  |  |  |  |
| None |  | 16 (64.0) |  |  |
| < 1 hour |  | 1 (4.0) |  |  |
| 1 – 4 hours |  | 0 (0.0) |  |  |
| 5 – 12 hours |  | 0 (0.0) |  |  |
| 13 – 24 hours |  | 2 (8.0) |  |  |
| Ongoing |  | 6 (24.0) |  |  |
| For how many **days** did the swelling/redness last? |  |  |  |  |
| None |  |  | 16 (64.0) | 21 (84.0) |
| < 1 day |  |  | 1 (4.0) | 2 (8.0) |
| 1 – 2 days |  |  | 3 (12.0) | 1 (4.0) |
| 3 – 4 days |  |  | 4 (16.0) | 1 (4.0) |
| 5 – 7 days |  |  | 1 (4.0) | 0 (0.0) |
| Ongoing |  |  | 0 (0.0) | 0 (0.0) |
| Please rate the level of any ***lasting pain*** your child felt since he/she received the vaccine patch, from 0 (no lasting pain) to 10 (lasting pain as bad as you can imagine)^2^ | 0.1 (0.4) | 0.0 (0.0) | 0.0 (.0.0) | 0.0 (0.0) |
| Please rate the level of any ***swelling and/or redness at the application site*** your child experienced since he/she received the vaccine patch, from 0 (no swelling and/or redness at the site) to 10 (swelling and/or redness as bad as you can imagine)^2^ | 1.3 (1.3) | 0.8 (1.4) | 0.6 (0.9) | 0.5 (1.2) |
| Compared to **vaccine *shot*,** was receiving the vaccine patch a better or worse experience? *^,1^ |  |  |  |  |
| Better than shot | 23  (92.0) | 24  (92.3) | 20  (76.9) | 24  (96.0) |
| Neither better nor worse | 1  (4.0) | 1  (3.9) | 3  (11.5) | 1  (4.0) |
| Worse | 0  (0.0) | 0  (0.0) | 0  (0.0) | 0  (0.0) |
| Don’t Know | 0  (0.0) | 0  (0.0) | 0  (0.0) | 0  (0.0) |
| Child never had shot | 1  (4.0) | 1  (3.9) | 3  (11.5) | 0  (0.0) |
| Compared to a vaccine ***nasal spray*,** was receiving the vaccine patch a better or worse experience? *^,1^ |  |  |  |  |
| Better than spray | 8  (32.0) | 7  (28.0) | 8  (32.0) | 4  (16.0) |
| Neither better nor worse | 2  (8.0) | 1  (4.0) | 1  (4.0) | 1  (4.0) |
| Worse | 0  (0.0) | 0  (0.0) | 0  (0.0) | 0  (0.0) |
| Don’t know | 2  (8.0) | 1  (4.0) | 0  (0.0) | 0  (0.0) |
| Child never had spray | 13  (52.0) | 16  (64.0) | 16  (64.0) | 20  (80.0) |

*Participants could select multiple options

**Discrepancies in number of responses for survey questions in the different columns varies based on parent/LAR response/lack of response. For single-answer survey questions, the maximum number of answers for Day 15 is 22, all other Days the maximum number of answers is 25

^1^ Results reported as the number and corresponding percentage (in parentheses) of parents who selected a particular answer

^2^ Results reported as the mean score and standard deviation (in parentheses)

## **Supplementary Table 4: Parental Acceptability Survey Results: Perception of Placebo dMNP Experience (dMNP #2)**

| **Symptom** | **Post-dMNP**  **Day 8**** | **Post-dMNP Day 9** | **Post-dMNP Day 15^** | **Final Visit***** |
| --- | --- | --- | --- | --- |
| Since the time your child received a vaccine patch, has your child experienced any lasting pain resulting from the vaccine patch and for how long^1^: |  |  |  |  |
| For how many **minutes** did the lasting pain last? |  |  |  |  |
| None | 17 (73.9) |  |  |  |
| < 1 minute | 5 (21.7) |  |  |  |
| 1 – 5 minutes | 0 (0.0) |  |  |  |
| 6 – 10 minutes | 0 (0.0) |  |  |  |
| 11 – 30 minutes | 1 (4.3) |  |  |  |
| Ongoing | 0 (0.0) |  |  |  |
| For how many **hours** did the lasting pain last? |  |  |  |  |
| None |  | 23 (100.0) |  |  |
| < 1 hour |  | 0 (0.0) |  |  |
| 1 – 4 hours |  | 0 (0.0) |  |  |
| 5 – 12 hours |  | 0 (0.0) |  |  |
| 13 – 24 hours |  | 0 (0.0) |  |  |
| Ongoing |  | 0 (0.0) |  |  |
| For how many **days** did the lasting pain last? |  |  |  |  |
| None |  |  | 22 (100.0) | 23 (100.0) |
| < 1 day |  |  | 0 (0.0) | 0 (0.0) |
| 1 – 2 days |  |  | 0 (0.0) | 0 (0.0) |
| 3 – 4 days |  |  | 0 (0.0) | 0 (0.0) |
| 5 – 7 days |  |  | 0 (0.0) | 0 (0.0) |
| Ongoing |  |  | 0 (0.0) | 0 (0.0) |
| For how many **minutes** did the swelling/redness last? |  |  |  |  |
| None | 9 (39.1) |  |  |  |
| < 1 minute | 2 (8.7) |  |  |  |
| 1 – 5 minutes | 1 (4.3) |  |  |  |
| 6 – 10 minutes | 0 (0.0) |  |  |  |
| 11 – 30 minutes | 4 (17.4) |  |  |  |
| Ongoing | 7 (30.4) |  |  |  |
| For how many **hours** did the swelling/redness last? |  |  |  |  |
| None |  | 14 (60.9) |  |  |
| < 1 hour |  | 2 (8.7) |  |  |
| 1 – 4 hours |  | 0 (0.0) |  |  |
| 5 – 12 hours |  | 1 (4.3) |  |  |
| 13 – 24 hours |  | 2 (8.7) |  |  |
| Ongoing |  | 4 (17.4) |  |  |
| For how many **days** did the swelling/redness last? |  |  |  |  |
| None |  |  | 15 (68.2) | 21 (91.3) |
| < 1 day |  |  | 4 (18.2) | 2 (8.7) |
| 1 – 2 days |  |  | 1 (45.5) | 0 (0.0) |
| 3 – 4 days |  |  | 2 (9.1) | 0 (0.0) |
| 5 – 7 days |  |  | 0 (0.0) | 0 (0.0) |
| Ongoing |  |  | 0 (0.0) | 0 (0.0) |
| Please rate the level of any ***lasting pain*** your child felt since he/she received the vaccine patch, from 0 (no lasting pain) to 10 (lasting pain as bad as you can imagine)^2^ | 0.3 (0.5) | 0.0 (0.0) | 0.0 (0.0) | 0.0 (0.0 |
| Please rate the level of any ***swelling and/or redness at the application site*** your child experienced since he/she received the vaccine patch, from 0 (no swelling and/or redness at the site) to 10 (swelling and/or redness as bad as you can imagine)^2^ | 1.4 (1.8) | 0.5 (0.7) | 0.4 (0.7) | 0.2 (0.5) |
| Compared to **vaccine *shot*,** was receiving the vaccine patch a better or worse experience? ^*,2^ |  |  |  |  |
| Better than shot | 18  (78.3) | 19  (82.6) | 19  (86.4) | 22  (95.7) |
| Neither better nor worse | 4  (17.4) | 2  (8.7) | 1  (4.5) | 1  (4.3) |
| Worse | 0  (0.0) | 1  (4.3) | 0  (0.0) | 0  (0.0) |
| Don’t Know | 0  (0.0) | 0  (0.0) | 0  (0.0) | 0  (0.0) |
| Child never had shot | 1  (4.3) | 1  (4.3) | 2  (9.1) | 0  (0.0) |
| Compared to a vaccine ***nasal spray*,** was receiving the vaccine patch a better or worse experience? ^*,2^ |  |  |  |  |
| Better than spray | 3  (13.0) | 8  (33.3) | 6  (26.1) | 4  (17.4) |
| Neither better nor worse | 4  (17.4) | 1  (4.2) | 0  (0.0) | 1  (4.3) |
| Worse | 0  (0.0) | 0  (0.0) | 0  (0.0) | 0  (0.0) |
| Don’t know | 0  (0.0) | 0  (0.0) | 0  (0.0) | 0  (0.0) |
| Child never had spray | 16  (69.6) | 15  (62.5) | 17  (73.9) | 18  (78.3) |

*Participants could select multiple options

^One participant did not attend Visit 5 (Day 15)

**Discrepancies in number of responses for survey questions in the different columns varies based on parent/LAR response/lack of response. For single-answer survey questions, the maximum number of answers for Day 15 is 22, all other Days the maximum number of answers is 25

^1^ Results reported as the number and corresponding percentage (in parentheses) of parents who selected a particular answer

^2^ Results reported as the mean score and standard deviation (in parentheses)

## **Supplementary Table 5: Parental Acceptability Survey Results: Perception of Placebo dMNP Experience (dMNP #3)**

| **Symptom** | **Post-dMNP Day 8** | **Post-dMNP Day 9** | **Post-dMNP Day 15^** | **Final Visit***** |
| --- | --- | --- | --- | --- |
| Since the time your child received a vaccine patch, has your child experienced any lasting pain resulting from the vaccine patch and for how long^1^: |  |  |  |  |
| For how many **minutes** did the lasting pain last? |  |  |  |  |
| None | 17 (73.9) |  |  |  |
| < 1 minute | 5 (21.7) |  |  |  |
| 1 – 5 minutes | 0 (0.0) |  |  |  |
| 6 – 10 minutes | 0 (0.0) |  |  |  |
| 11 – 30 minutes | 1 (4.3) |  |  |  |
| Ongoing | 0 (0.0) |  |  |  |
| For how many **hours** did the lasting pain last? |  |  |  |  |
| None |  | 23 (100.0) |  |  |
| < 1 hour |  | 0 (0.0) |  |  |
| 1 – 4 hours |  | 0 (0.0) |  |  |
| 5 – 12 hours |  | 0 (0.0) |  |  |
| 13 – 24 hours |  | 0 (0.0) |  |  |
| Ongoing |  | 0 (0.0) |  |  |
| For how many **days** did the lasting pain last? |  |  |  |  |
| None |  |  | 22 (100.0) | 23 (100.0) |
| < 1 day |  |  | 0 (0.0) | 0 (0.0) |
| 1 – 2 days |  |  | 0 (0.0) | 0 (0.0) |
| 3 – 4 days |  |  | 0 (0.0) | 0 (0.0) |
| 5 – 7 days |  |  | 0 (0.0) | 0 (0.0) |
| Ongoing |  |  | 0 (0.0) | 0 (0.0) |
| For how many **minutes** did the swelling/redness last? |  |  |  |  |
| None | 7 (30.4) |  |  |  |
| < 1 minute | 2 (8.7) |  |  |  |
| 1 – 5 minutes | 2 (8.7) |  |  |  |
| 6 – 10 minutes | 0 (0.0) |  |  |  |
| 11 – 30 minutes | 4 (17.4) |  |  |  |
| Ongoing | 8 (34.8) |  |  |  |
| For how many **hours** did the swelling/redness last? |  |  |  |  |
| None |  | 14 (60.9) |  |  |
| < 1 hour |  | 2 (8.7) |  |  |
| 1 – 4 hours |  | 0 (0.0) |  |  |
| 5 – 12 hours |  | 2 (8.7) |  |  |
| 13 – 24 hours |  | 2 (8.7) |  |  |
| Ongoing |  | 3 (13.0) |  |  |
| For how many **days** did the swelling/redness last? |  |  |  |  |
| None |  |  | 15 (68.2) | 21 (91.3) |
| < 1 day |  |  | 5 (22.7) | 2 (8.7) |
| 1 – 2 days |  |  | 1 (4.5) | 0 (0.0) |
| 3 – 4 days |  |  | 1 (4.5) | 0 (0.0) |
| 5 – 7 days |  |  | 0 (0.0) | 0 (0.0) |
| Ongoing |  |  | 0 (0.0) | 0 (0.0) |
| Please rate the level of any ***lasting pain*** your child felt since he/she received the vaccine patch, from 0 (no lasting pain) to 10 (lasting pain as bad as you can imagine)^2^ | 0.3 (0.5) | 0.3 (0.5) | 0.0 (0.0) | 0.0 (0.0) |
| Please rate the level of any ***swelling and/or redness at the application site*** your child experienced since he/she received the vaccine patch, from 0 (no swelling and/or redness at the site) to 10 (swelling and/or redness as bad as you can imagine)^2^ | 1.4 (1.7) | 0.5 (0.7) | 0.4 (0.7) | 0.2 (0.5) |
| Compared to **vaccine *shot*,** was receiving the vaccine patch a better or worse experience? *^,1^ |  |  |  |  |
| Better than shot | 18  (78.3) | 17  (73.9) | 18  (81.8) | 21  (91.3) |
| Neither better nor worse | 4  (17.4) | 3  (13.0) | 2  (9.1) | 2  (8.7) |
| Worse | 0  (0.0) | 1  (4.3) | 0  (0.0) | 0  (0.0) |
| Don’t Know | 0  (0.0) | 0  (0.0) | 0  (0.0) | 0  (0.0) |
| Child never had shot | 1  (4.3) | 2  (8.7) | 2  (9.1) | 0  (0.0) |
| Compared to a vaccine ***nasal spray*,** was receiving the vaccine patch a better or worse experience? *^,1^ |  |  |  |  |
| Better than spray | 3  (13.0) | 8  (33.3) | 6  (26.1) | 4  (17.4) |
| Neither better nor worse | 4  (17.4) | 1  (4.2) | 0  (0.0) | 1  (4.3) |
| Worse | 0  (0.0) | 0  (0.0) | 0  (0.0) | 0  (0.0) |
| Don’t know | 0  (0.0) | 0  (0.0) | 0  (0.0) | 0  (0.0) |
| Child never had spray | 16  (69.6) | 15  (62.5) | 17  (73.9) | 18  (78.3) |

*Participants could select multiple options

^One participant did not attend Visit 5 (Day 15)

**Discrepancies in number of responses for survey questions varies based on parent/LAR response/lack of response. For single-answer survey questions, the maximum number of answers for Day 15 is 22, all other Days the maximum number of answers is 25

^1^ Results reported as the number and corresponding percentage (in parentheses) of parents who selected a particular answer

^2^ Results reported as the mean score and standard deviation (in parentheses)

## **Supplementary Table 6: Parental Acceptability Survey Results: Perception of Placebo dMNP Experience at Final Study Visit (dMNPs #1, #2, #3)**

|  | **Final Study Visit** | | |
| --- | --- | --- | --- |
| **Symptom** | **dMNP #1** | **dMNP #2** | **dMNP #3** |
| Since the time your child received a vaccine patch, has your child experienced any lasting pain resulting from the vaccine patch and for how long^1^: |  |  |  |
| For how many **days** did the lasting pain last? |  |  |  |
| None | 25 (100.0) | 23 (100.0) | 23 (100.0) |
| < 1 day | 0 (0.0) | 0 (0.0) | 0 (0.0) |
| 1 – 2 days | 0 (0.0) | 0 (0.0) | 0 (0.0) |
| 3 – 4 days | 0 (0.0) | 0 (0.0) | 0 (0.0) |
| 5 – 7 days | 0 (0.0) | 0 (0.0) | 0 (0.0) |
| Ongoing | 0 (0.0) | 0 (0.0) | 0 (0.0) |
| For how many **days** did the swelling/redness last? |  |  |  |
| None | 21 (84.0) | 21 (91.3) | 21 (91.3) |
| < 1 day | 2 (8.0) | 2 (8.7) | 2 (8.7) |
| 1 – 2 days | 1 (4.0) | 0 (0.0) | 0 (0.0) |
| 3 – 4 days | 1 (4.0) | 0 (0.0) | 0 (0.0) |
| 5 – 7 days | 0 (0.0) | 0 (0.0) | 0 (0.0) |
| Ongoing | 0 (0.0) | 0 (0.0) | 0 (0.0) |
| Please rate the level of any ***lasting pain*** your child felt since he/she received the vaccine patch, from 0 (no lasting pain) to 10 (lasting pain as bad as you can imagine)^2^ | 0.0 (0.0) | 0.0 (0.0 | 0.0 (0.0) |
| Please rate the level of any ***swelling and/or redness at the application site*** your child experienced since he/she received the vaccine patch, from 0 (no swelling and/or redness at the site) to 10 (swelling and/or redness as bad as you can imagine)^2^ | 0.5 (1.2) | 0.2 (0.5) | 0.2 (0.5) |
| Compared to **vaccine *shot*,** was receiving the vaccine patch a better or worse experience? *^,1^ |  |  |  |
| Better than shot | 24  (96.0) | 22  (95.6) | 21  (91.3) |
| Neither better nor worse | 1  (4.0) | 1  (4.4) | 2  (8.7) |
| Worse | 0  (0.0) | 0  (0.0) | 0  (0.0) |
| Don’t Know | 0  (0.0) | 0  (0.0) | 0  (0.0) |
| Child never had shot | 0  (0.0) | 0  (0.0) | 0  (0.0) |
| Compared to a vaccine ***nasal spray*,** was receiving the vaccine patch a better or worse experience? *^,1^ |  |  |  |
| Better than spray | 4  (16.0) | 4  (17.4) | 4  (17.4) |
| Neither better nor worse | 1  (4.0) | 1  (4.3) | 1  (4.3) |
| Worse | 0  (0.0) | 0  (0.0) | 0  (0.0) |
| Don’t know | 0  (0.0) | 0  (0.0) | 0  (0.0) |
| Child never had spray | 20  (80.0) | 18  (78.3) | 18  (78.3) |

*Participants could select multiple options

**Discrepancies in number of responses for survey questions varies based on parent/LAR response/lack of response. For single-answer survey questions, then maximum number of answers is 25

^1^ Results reported as the number and corresponding percentage (in parentheses) of parents who selected a particular answer

^2^ Results reported as the mean score and standard deviation (in parentheses)

## **Supplementary Table 7: Overall Experience with the Placebo dMNP**

| **How do you feel about the overall experience so far: (1 – 5)*** | **n** | **Mean** | **Median** | **Standard Deviation** | **Min** | **Max** |
| --- | --- | --- | --- | --- | --- | --- |
| dMNP #1, Day 1 | 25 | 4.5 | 5 | 0.6 | 3 | 5 |
| dMNP #1, Day 2 | 25 | 4.6 | 5 | 0.5 | 4 | 5 |
| dMNP #1, Day 8 | 25 | 4.6 | 5 | 0.7 | 3 | 5 |
| dMNP #2/3, Day 8 | 23 | 4.4 | 5 | 0.8 | 3 | 5 |
| dMNP #2/3, Day 9 | 22** | 4.7 | 5 | 0.6 | 3 | 5 |
| dMNP #2/3, Day 15 | 22** | 4.7 | 5 | 0.5 | 4 | 5 |
| dMNP #1/2/3, End of study | 25 | 4.8 | 5 | 0.4 | 4 | 5 |
| dMNP #1: n = 25  dMNP #2/3: n = 23  *1 to 5 scale (1 – Very Negative, 2 – Negative, 3 – Neutral, 4 – Positive, 5 – Very positive)  **1 participant who received dMNP #2/3 missed visit Day 9 & 15, n=22 | | | | | | |
